# Different effects of partial pressure in a high-pressure gaseous mixture of carbon monoxide and oxygen for rat heart preservation

**DOI:** 10.1038/s41598-019-43905-0

**Published:** 2019-05-16

**Authors:** Naoyuki Hatayama, Shuichi Hirai, Kaori Fukushige, Hiroki Yokota, Masahiro Itoh, Munekazu Naito

**Affiliations:** 1 Department of Anatomy, Aichi Medical University, Aichi, Japan; 2 Department of Anatomy, Tokyo Medical University, Tokyo, Japan

**Keywords:** Experimental models of disease, Heart failure

## Abstract

We maintained the function of an extracted rat heart after 24–48 h preservation in a high-pressure gaseous mixture of carbon monoxide (CO) and oxygen (O_2_). Here, we assessed the effects of different partial pressures of hyperbaric CO and O_2_ for 24–48 h at 4 °C on rat heart preservation and compared conditions including immersion in University of Wisconsin solution. Preserved hearts were transplanted into recipient rats via heterotopic cervical heart transplantation for *in vivo* evaluation and perfused using the Langendorff system for *ex vivo* evaluation. The survival rate of transplanted hearts was 100% at postoperative day 7 in the CO + O_2_ (PCO:PO_2_ = 1.5:2.0 atm) group but only 33% in the CO + O_2_ (PCO:PO_2_ = 2.0:1.5 atm) group. Langendorff system and histopathological analysis revealed that the left ventricular pressure of preserved hearts in the CO + O_2_ (PCO:PO_2_ = 1.5:2.0 atm) group was better than the CO + O_2_ (PCO:PO_2_ = 2.0:1.5 atm). We demonstrate that exposure of rat hearts to hyperbaric CO and O_2_ is superior to the immersion method and that partial pressure of hyperbaric CO and O_2_ is crucial to preservation.

## Introduction

Carbon monoxide (CO) is regarded as poisonous because of its high affinity for haemoglobin that causes rapid elevation of carboxyhemoglobin to toxic levels that compromise oxygen delivery to the tissues^1,2^. On the other hand, CO exerts vasoactive, antiproliferative, antioxidant, anti-inflammatory, and antiapoptotic effects and significantly contributes to cell protection^3^. Studies have suggested a beneficial effect of gaseous CO under pathophysiological conditions, such as organ transplantation, ischaemia reperfusion injury (IRI), inflammation, sepsis, or shock states^3,4^. However, hyperbaric O_2_ treatment has been shown to damage lung function, potentially causing cellular dysfunction. Clinically, hyperbaric O_2_ is only used in limited circumstances, such as after heart surgery, and extended periods of treatment are usually avoided. On the other hand, hyperbaric O_2_ can reduce IRI that worsens crush injuries, induce the compartment syndrome, and cause skin flap and reattachment failures by inhibiting the adherence of neutrophils that release proteases and produce free radicals^5,6^.

Currently, the preservation time for clinically extracted hearts prior to transplantation is 4–6 h^7^. Typically, preservation of a donor organ involves flushing and cooling the organ with a cold storage solution to remove blood components, then immersing it in a cold storage solution, in a process known as static cold storage (CS)^8,9^. CS solutions, such as University of Wisconsin (UW) and histidine–tryptophan–ketoglutarate solutions, have been developed to improve the quality of preserved organs and extend the acceptable preservation time limits for transplantation^10^. Recent studies have demonstrated the advantages of machine perfusion over CS preservation for kidney and liver transplantation^11,12^. Thus, the specific preservation method needs according to each organ characteristics and the original method should be developed for each organ.

We developed a high-pressure gas (HPG) preservation method using a mixture of CO and O_2_ gases and succeeded in resuscitating extracted rat hearts following 48 h of preservation under conditions of CO + O_2_ (PCO:PO_2_ = 4:3 atm)^13^. It was also shown that rat heart function after 24 h of high-pressure preservation using a mixture of CO and O_2_ gases was almost the same as control hearts^14^. Moreover, we reported successful transplantation of a rat limb following 7 days of preservation under conditions of CO + O_2_ (PCO:PO_2_ = 4:3 atm)^15^. We also showed significantly lower blood levels of creatine phosphokinase in recipients who received limbs preserved in a chamber containing CO and O_2_ gases for 18 h after wrapped in saline-moistened gauze for 6 h compared with limbs only wrapped in saline-moistened gauze for 24 h^15^. It was also reported that hyperbaric CO and O_2_ successfully preserved rat kidney grafts for 24 h by protecting tubular epithelial cells from apoptosis and inhibiting inflammation^16^. Surprisingly, no protective effect was observed under conditions of CO + O_2_ (PCO:PO_2_ = 4:3 atm) compared with preservation of a rat kidney for 24 h in a chamber filled with air (1 atm)^16^. Therefore, we selected a lower partial pressure mixed gas composed of CO + O_2_ (PCO:PO_2_ = 2.0:1.5 atm) for kidney graft and found an improved graft survival^16^. In this way, the appropriate total and partial pressures differ for each organ, and the partial pressures of CO and O_2_ for HPG remain unknown. This study aimed to evaluate the difference of partial pressure between CO and O_2_ on HPG in rat hearts.

## Results

### Preservation methods in this study

**Figure 1:**
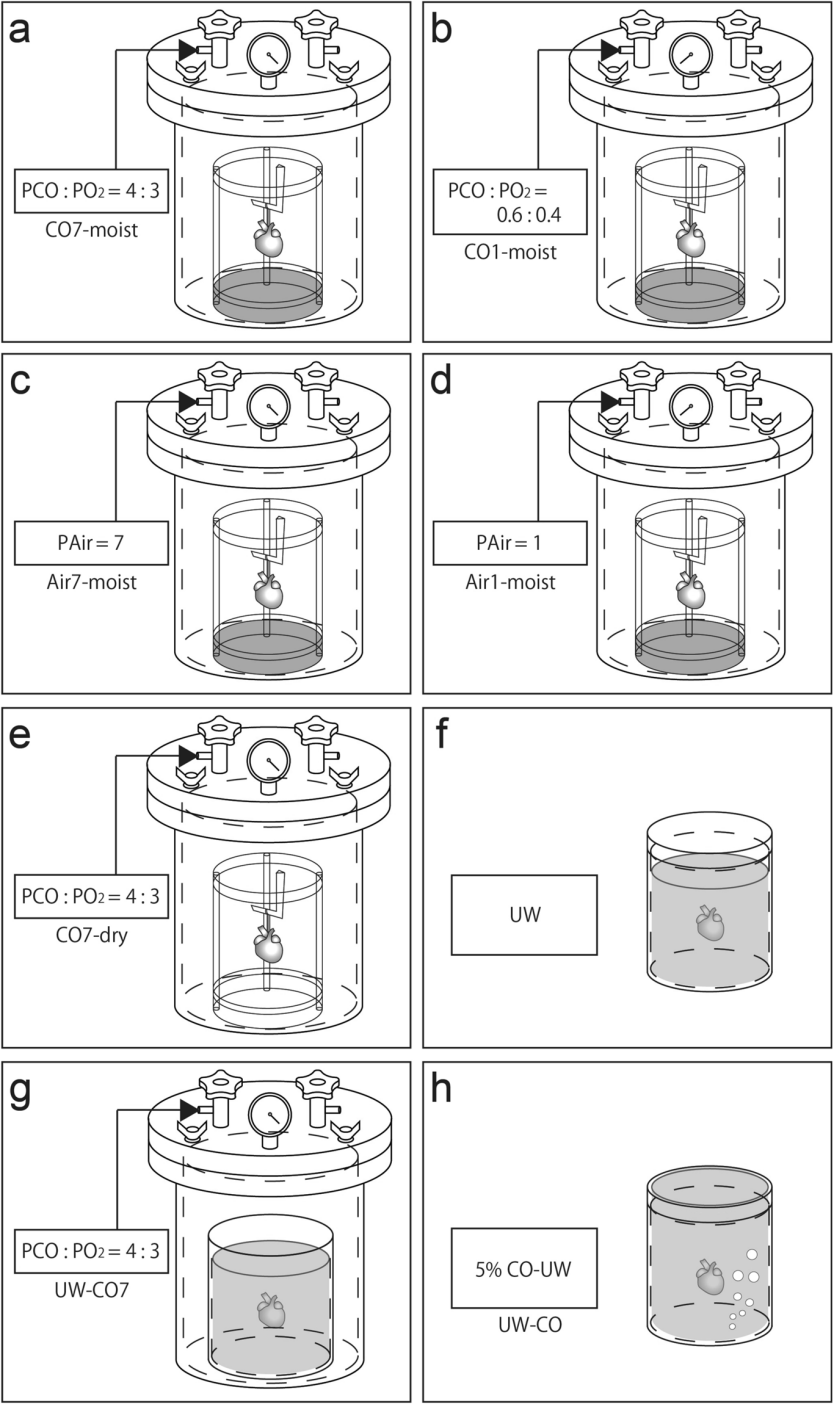
Conditions during rat heart extraction. (**a**) CO7-moist group, (**b**) CO1-moist group, (**c**) Air7-moist group, (**d**) Air1-moist group, (**e**) CO7-dry group, (**f**) UW-group, (**g**) UW-CO7 group, and (**h**) UW-CO group. CO, carbon monoxide; UW, University of Wisconsin solution.

HPG preservation requires specific conditions including pressure, humidity, medical gases, and gas phase. To verify the importance of these conditions for HPG preservation, hearts were extracted from rats and preserved for 48 h at 4 °C under eight different conditions: (1) chamber CO7-moisit group (Fig. 1a), hearts were hung inside a chamber filled with a mixture of CO and O_2_ gas (7 atm; PCO:PO_2_ = 4: 3 atm) and a flask containing 50 mL of distilled water was placed inside to maintain humidity; (2) CO1-moisit group (Fig. 1b), hearts were hung inside a chamber filled with a mixture of CO and O_2_ gas (1 atm; PCO:PO_2_ = 0.6:0.4 atm) with a flask with 50 mL of distilled water; (3) Air7-moisit group (Fig. 1c), hearts were hung inside a chamber filled with 7 atm air with a flask with 50 mL of distilled water; (4) Air1-moisit group (Fig. 1d), hearts were hung inside a chamber filled with 1 atm air with a flask with 50 mL of distilled water; (5) CO7-dry group (Fig. 1e), hearts were hung inside a chamber filled with a mixture of CO and O_2_ gas (7 atm; PCO:PO_2_ = 4:3 atm) without distilled water; (6) UW-group (Fig. 1f), hearts were immersed in UW solution (Viaspan, Du Pont, Wilmington, DE, USA); (7) UW-CO7 group (Fig. 1g), hearts were immersed in UW solution then placed in a chamber filled with a mixture of CO and O_2_ gas (7 atm; PCO:PO_2_ = 4:3 atm); and (8) UW-CO group (Fig. 1h), hearts were immersed in UW solution into which CO had been dissolved according to the method described by Kohmoto *et al*.^17^. Briefly, UW solution was vigorously bubbled at 4 °C before use for 5 min with compressed 5% CO gas mixed in air. In order to maintain soluble CO in the UW solution, CO-bubbled and CO-equilibrated UW solution was kept in a tightly sealed container with a secured lid without an air layer. In all hearts, blood was removed using the Krebs–Henseleit solution (118 mM NaCl, 4.7 mM KCl, 1.8 mM CaCl_2_.2H_2_O, 1.2 mM MgSO4.7H_2_O, 1.2 mM NaH_2_PO_4_.2H_2_O, 25.0 mM NaHCO_3_, and 11.1 mM glucose) prior to preservation. Preserved hearts within UW solution were reinfused with UW solution after blood removal. In addition, control group was harvested hearts from donor rats were immediately transplanted to the recipient rats without preservation.

### Chamber humidity and changes of heart weight before and after 48 h of preservation using different methods

Chamber humidity and changes of heart weight before and after preservation in each group are shown in Table 1. The humidity in the CO7-dry group was significantly lower than that in the CO7-moist group (61.0 ± 1% vs 96.3 ± 1.06%, *P* < 0.05). In the other groups, the humidity was approximately 96% and was not significantly different compared with the CO7-moist group. The rate of change in the CO7-dry group was significantly higher than that of the CO7-moist group (−19.2 ± 5.43% vs −13.4 ± 1.77%, *P* < 0.05). The heart weights in UW, UW-CO7, and UW-CO groups decreased (by approximately 15%) after preservation, and the decrease in these groups was greater than that in the CO7-moist group.

**Table 1:** Humidity in the chamber, change of heart weight before and after 48 h of preservation, and revival and survival rates of post-transplanted hearts using different preservation methods.

Group	Preservation method	Humidity in the chamber (%)	n	Heart weight			Revival rate (%)	Survival rate	
				before preservation (g)	after preservation (g)	rate of change (%)		at 1 hour (%)	on day 7 (%)
CO 7 -moist	Fig. 1a	96.3 ± 1.06^a^	12	1.52 ± 0.27	1.31 ± 0.22^b^	−13.40 ± 1.77^c^	12/12 (100)	12/12 (100)	9/12 (75)
CO 1 -moist	Fig. 1b	96.3 ± 1.51	6	1.48 ± 0.24	1.28 ± 0.22	−13.85 ± 1.63	5/6 (83)	2/6 (33)	0/6 (0)
Air 7 -moist	Fig. 1c	96.4 ± 1.14	5	1.49 ± 0.25	1.30 ± 0.21	−13.20 ± 1.51	2/5 (40)	0/5 (0)	0/5 (0)
Air 1 -moist	Fig. 1d	96.1 ± 1.33	5	1.51 ± 0.24	1.33 ± 0.23	−11.87 ± 2.25	0/5 (0)	0/5 (0)	0/5 (0)
CO 7 -dry	Fig. 1e	61.0 ± 1.00^d^	5	1.49 ± 0.31	1.19 ± 0.19^e^	−19.20 ± 5.43^f^	4/5 (80)	2/5 (40)	0/5 (0)
UW	Fig. 1f	−	5	1.42 ± 0.41	1.20 ± 0.35^g^	−15.12 ± 2.11^h^	0/5 (0)	0/5 (0)	0/5 (0)
UW -CO7	Fig. 1g	96.2 ± 1.10	6	1.44 ± 0.30	1.22 ± 0.25	−15.33 ± 2.06	0/6 (0)	0/6 (0)	0/6 (0)
UW -CO	Fig. 1h	—	5	1.45 ± 0.21	1.23 ± 0.16	−15.12 ± 2.77	0/5 (0)	0/5 (0)	0/5 (0)
Control							8/8 (100)	8/8 (100)	8/8 (100)

Values are expressed as the mean SD. P < 0.05: a vs d, b vs e, g, c vs f, h.

P-values were calculated by one-way ANOVA with post hoc Tukey’s multiple comparison analysis.

### Revival and survival rates of transplanted hearts after 48 h of preservation using different methods

The revival and survival rates of transplanted hearts from each group are shown in Table 1. After the heterotopic cervical heart transplantation, the revival rate of post-transplanted hearts was 100% (12/12) in the CO7-moist and control groups, 80% (4/5) in CO7-dry group, 40% (2/5) in the Air7-moist group, and 83% (5/6) in the CO1-moist group but was 0% (0/5) in the Air1-moist group. All post-transplanted hearts from the three groups in which the extracted hearts were immersed in preservation solutions (UW, UW-CO7, and UW-CO groups) could not be resuscitated. The survival rate of the transplanted hearts on postoperative day 7 was 100% (8/8) in the control group and 75% (9/12) in the CO7-moist groups, but 0% in the other groups.

### Revival and survival rates of transplanted hearts after 48 h of preservation under different partial pressures of CO-moist using HPG

The revival and survival rates of transplanted hearts after preservation under different partial pressures of CO and O_2_ are shown in Table 2. After heterotopic cervical heart transplantation, the revival rate of post-transplanted hearts after perseveration with CO + O_2_ (PCO:PO_2_ = 4:3 atm) was 100% (12/12), whereas this was 67% (6/9) for CO + O_2_ (PCO:PO_2_ = 3:4 atm) and 33% (2/6) for CO + O_2_ (PCO:PO_2_ = 3.5:3.5 atm). The post-transplanted hearts preserved using CO (PCO = 7 atm) or O_2_ (PO_2_ = 7 atm) could not be resuscitated. The survival rate of post-transplanted hearts on postoperative day 7 after perseveration using CO + O_2_ (PCO:PO_2_ = 4:3 atm) was 75% (9/12), whereas this was 33% (3/9) using CO + O_2_ (PCO:PO_2_ = 3:4 atm).

**Table 2:** Revival and survival rates of transplanted hearts after 48 h of preservation using different partial pressures of CO and O_2_.

Group	Partial pressure CO:O2 (atm)	n	Revival rate (%)	Survival rate on day 7 (%)
CO 7 -moist	7: 0	5	0/5 (0)	0/5 (0)
	4: 3	12	12/12 (100)	9/12 (75)
	3.5: 3.5	6	2/6 (33)	0/6 (0)
	3: 4	9	6/9 (67)	3/9 (33)
	0: 7	5	0/5 (0)	0/5 (0)
CO 3.5 -moist	3.5: 0	5	0/5 (0)	0/5 (0)
	2.0: 1.5	9	6/9 (67)	3/9 (33)
	1.75: 1.75	8	4/8 (50)	2/8 (25)
	1.5: 2.0	12	12/12 (100)	12/12 (100)
	0: 3.5	5	0/5 (0)	0/5 (0)
CO 1 -moist	1: 0	5	0/5 (0)	0/5 (0)
	0.6: 0.4	8	1/8 (13)	0/8 (0)
	0.5: 0.5	8	1/8 (13)	0/8 (0)
	0.4: 0.6	8	2/8 (25)	0/8 (0)
	0: 1	5	0/5 (0)	0/5 (0)

The revival rate of post-transplanted hearts after perseveration was 67% (6/9) using CO + O_2_ (PCO:PO_2_ = 2.0:1.5 atm), 50% (4/8) using CO + O_2_ (PCO:PO_2_ = 1.75:1.75 atm), and 100% (12/12) using CO + O_2_ (PCO:PO_2_ = 1.5:2.0 atm). All post-transplanted hearts preserved using CO (PCO = 3.5 atm) or O_2_ (PO_2_ = 3.5 atm) could not be resuscitated. The survival rate of post-transplanted hearts at postoperative day 7 was 33% (3/9) after perseveration using CO + O_2_ (PCO:PO_2_ = 2.0:1.5 atm) and 25% (2/8) using CO (PCO:PO_2_ = 1.75:1.75 atm), whereas this was 100% (12/12) using CO + O_2_ (PCO:PO_2_ = 1.5:2.0 atm).

The revival rate of post-transplanted hearts was 13% (1/8) after perseveration using CO + O_2_ (PCO:PO_2_ = 0.6:0.4 atm), 13% (1/8) using CO + O_2_ (PCO:PO_2_ = 0.5:0.5 atm), and 25% (2/8) using CO + O_2_ (PCO:PO_2_ = 0.4:0.6 atm). Post-transplanted hearts could not be resuscitated after preservation using CO (PCO = 1 atm) or O_2_ (PO_2_ = 1 atm).

### Comparison of cardiac function after 24 h of preservation under conditions between CO + O_2_ (PCO:PO_2_ = 1.5:2.0 atm) and CO + O_2_ (PCO:PO_2_ = 2.0:1.5 atm) with heterotopic cervical heart Transplantation

**Figure 2:**
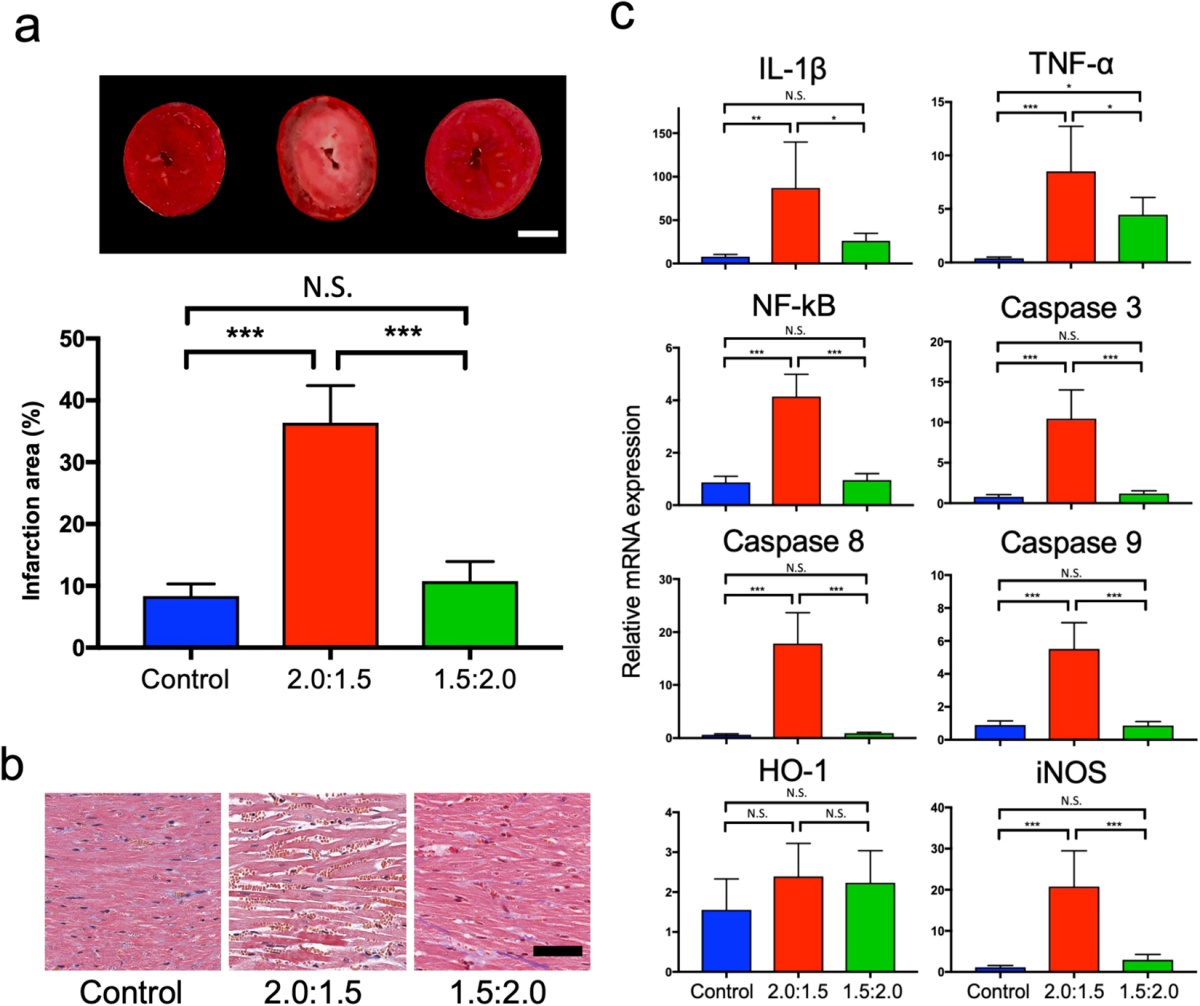
Cardiac function after 24 h of preservation under conditions between CO + O_2_ (PCO:PO_2_ = 2.0:1.5 atm) and CO + O_2_ (PCO:PO_2_ = 1.5:2.0 atm) using heterotopic cervical heart transplantation. (**a**) Representative mid-myocardial cross sections of TTC-stained hearts for the three groups (n = 6 for each group). Red-stained areas indicate viable tissue, and white areas indicate infarct tissue. White bar represents 500 μm. Myocardial infarction areas in the hearts from the three groups were quantified. *P* < 0.05 was considered statistically significant. N.S., not significant. ***P < 0.001. (**b**) Masson’s trichrome staining in the hearts from the three groups. Black bar represents 50 μm. (**c**) mRNA expression levels measured by quantitative RT-PCR in grafted hearts (n = 6 for each group). N.S., not significant. *P < 0.05, **P < 0.01, ***P < 0.001. *P*-values were calculated by one-way ANOVA with post hoc Tukey’s multiple comparison analysis.

Measurement of the myocardial infarct area using 2,3,5-triphenyltetrazolium chloride (TTC) staining was significantly greater in the CO + O_2_ (PCO:PO_2_ = 2.0:1.5 atm) group (36.4% ± 5.96) compared with the control (8.4% ± 1.96) and CO + O_2_ (PCO:PO_2_ = 1.5:2.0 atm) (10.8% ± 3.16) groups (*P* < 0.001 for both). However, there was no significant difference between the control and CO + O_2_ (PCO:PO_2_ = 1.5:2.0 atm) groups (Fig. 2a).

Light microscopy revealed that the myocardium in the CO + O_2_ (PCO:PO_2_ = 1.5:2.0 atm) group maintained almost normal tissue structure and shape, with the myocardial fibres arranged in an orderly manner (Fig. 2b). However, the CO + O_2_ (PCO:PO_2_ = 2.0:1.5 atm) group showed acute reperfusion injury characterised by haemorrhage (Fig. 2b).

Real-time reverse transcriptase PCR analyses revealed no changes in the expression of heme oxygenase-1 (HO-1) mRNA among the CO + O_2_ (PCO:PO_2_ = 2.0:1.5 atm), CO + O_2_ (PCO:PO_2_ = 1.5:2.0 atm), and control groups (Fig. 2c). A large increase was noted in the mRNA expression of caspases 3, 8, and 9, and inducible nitric oxide synthase (iNOS), and nuclear factor-kappa B (NF-κB) in the CO + O_2_ (PCO:PO_2_ = 2.0:1.5 atm) group but not in the control and CO + O_2_ (PCO:PO_2_ = 1.5:2.0 atm) groups (Fig. 2c). Expression of tumour necrosis factor-alpha (TNF-α) significantly increased in the CO + O_2_ (PCO:PO_2_ = 2.0:1.5 atm) and CO + O_2_ (PCO:PO_2_ = 1.5:2.0 atm) groups but not in the control group. Expression of interleukin-1 beta (IL-1β) was higher in the CO + O_2_ (PCO:PO_2_ = 2.0:1.5 atm) group compared with the control and CO + O_2_ (PCO:PO_2_ = 1.5:2.0 atm) groups (Fig. 2c).

### Comparison of cardiac function after 6, 12, 18, 24, and 48 h of preservation under conditions between CO + O_2_ (PCO:PO_2_ = 1.5:2.0 atm) and CO + O_2_ (PCO:PO_2_ = 2.0:1.5 atm) using the Langendorff system

**Figure 3:**
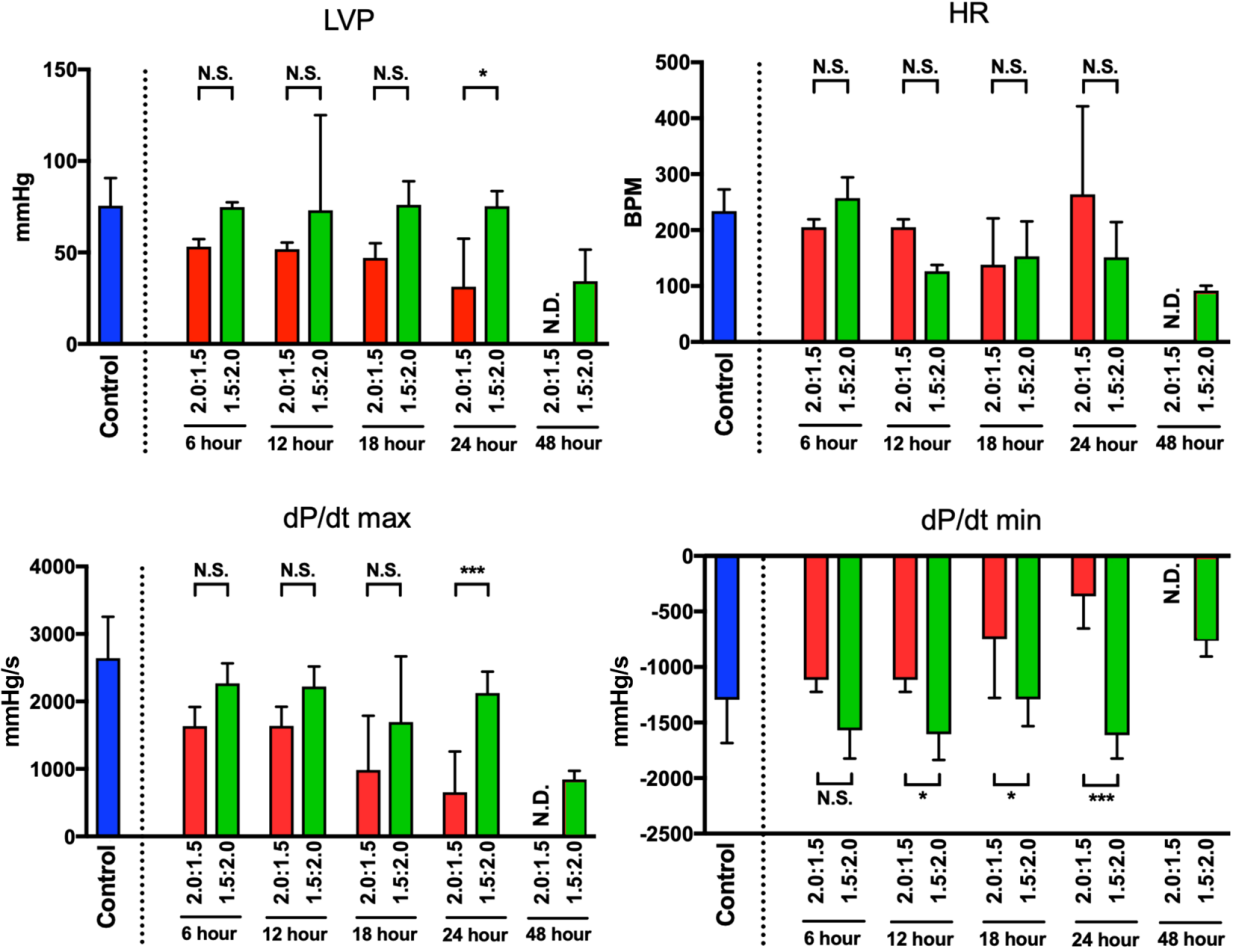
Cardiac function after 6, 12, 18, 24, and 48 h of preservation under conditions of CO + O_2_ (PCO:PO_2_ = 2.0:1.5 atm) and CO + O_2_ (PCO:PO_2_ = 1.5:2.0 atm) using the Langendorff system. Harvested hearts from control group rats were immediately perfused using the Langendorff system (n = 6). LVP, left ventricular pressure; HR, heart rate; dP/dt max and min, peak pressure increase and decrease; N.D., not detected; N.S., not significant, *P < 0.05, ***P < 0.001. *P*-values were calculated by two-way ANOVA with post hoc Tukey’s multiple comparison analysis.

The results from the Langendorff system are shown in Fig. 3. After 48 h of preservation, hearts could beat in the CO + O_2_ (PCO:PO_2_ = 1.5:2.0 atm) group but not in the CO + O_2_ (PCO:PO_2_ = 2.0:1.5 atm) group. There was no significant difference of left ventricular pressure (LVP) after 6, 12, and 18 h of preservation between the two groups, whereas LVP after 24 h of preservation was significantly higher in the CO + O_2_ (PCO:PO_2_ = 1.5:2.0 atm) group compared with the CO + O_2_ (PCO:PO_2_ = 2.0:1.5 atm) group. Heart rate after 6, 12, 18, and 24 h of preservation did not difference in the two groups. The ventricular pressure contractility (dP/dt) max after 6, 12, and 18 h of preservation did not differ between the two groups, whereas the dP/dt max after 24 h of preservation was significantly higher in the CO + O_2_ (PCO:PO_2_ = 1.5:2.0 atm) group compared with the CO + O_2_ (PCO:PO_2_ = 2.0:1.5 atm) group. On the other hand, the dP/dt min after 6 h of preservation did not differ between the two groups, whereas the dP/dt min after 12, 18, and 24 h of preservation was significantly lower in the CO + O_2_ (PCO:PO_2_ = 1.5:2.0 atm) group compared with the CO + O_2_ (PCO:PO_2_ = 2.0:1.5 atm) group.

## Discussion

HPG preservation methods are useful to maintain organ function. There have been some reports about the optimum gas combination of O_2_, CO, carbon dioxide, helium, and nitrogen for effective organ preservation. In the present study, we first demonstrated that exposure of rat hearts to hyperbaric CO and O_2_ was better than using the immersion method, and IRI after preservation under conditions of CO + O_2_ (PCO:PO_2_ = 1.5:2.0 atm) was lower than that of CO + O_2_ (PCO:PO_2_ = 2.0:1.5 atm).

Previous studies have demonstrated that the osmotic pressure of the solution is important for CS and machine perfusion^8–10^. UW solution has a high osmotic pressure to prevent tissue oedema and cell death and is suitable to preserve human hearts for 4–6 h^17^. Conversely, excessive drying was predicted during HPG preservation due to organ exposure to the gas phase. Therefore, we measured heart weights before and after preservation. Immersion of extracted hearts in UW solution for 48 h (UW, UW-CO7, and UW-CO groups in Table 1) reduced heart weights by approximately 15%, and the heart grafts could not be resuscitated. In the dry chamber with gases (humidity approximately 61%) (CO7-dry group), the weights of the preserved hearts were reduced by approximately 19%, and function was not maintained. On the other hand, in the moist chamber with the same combination of gases (humidity approximately 96%) (CO 7-moist group), the preserved rat hearts were slightly dehydrated, and the weight decreased by only 13%, and 100% (12/12) of the heart grafts could be resuscitated. These results indicate that exposure of rat hearts to hyperbaric CO and O_2_ is superior to the immersion method to preserve long-term cardiac function. While the dehydration worsens preservation state in HPG preservation method, therefore, preserved organs should be kept in humid conditions.

In the present study, we dissolved CO into UW solution according to a previous study and verified that heart grafts could not be resuscitated after 48 h of preservation (UW-CO group, 0/5 in Table 1)^18^. Furthermore, when the UW solution within the extracted hearts was under conditions of CO + O_2_ (PCO:PO_2_ = 4:3 atm), allowing CO and O_2_ gases to dissolve into solution to a greater extent than at 1 atm, heart grafts could not be resuscitated after 48 h of preservation (UW-CO7 group: 0/6 in Table 1). These results suggest that CS using the immersion method of UW solution is limited to obtain the cell-protective effect of these gases on heart preservation. Heart grafts in the UW solution were under ambient pressure; however, resuscitation in UW solution under a pressure of 7 atm (UW-CO7 group) was not better than that under 1 atm (UW-CO group). These results indicated that the pressure itself may not improve storage conditions. Moreover, the survival rates of heart grafts in gases less than 7 atm (CO7-moisit group) was improved compared with those under 1 atm (CO1-moisit group) (Table 2). Henry’s Law states that the amount of gas that is dissolved in a preserved organ under a pressure of 7 atm increases seven times more than that under 1 atm. It is thought that the different solubilities of solutions used for preserving hearts under pressure could be one of the reasons to extend preservation time on rat heart due to enhanced protective effects of CO and O_2_. Conversely, we have previously described the preservation of rat kidney for 24 h and found no protective effects under conditions of CO + O_2_ (PCO:PO_2_ = 4:3 atm, 7ATA), whereas the use of CO + O_2_ (PCO:PO_2_ = 2.0:1.5 atm, 3.5ATA) improved the survival rate of the kidney graft^16^. This study also showed that the survival rates at postoperative day 7 using 3.5ATA (PCO:PO_2_ = 2.0:1.5 atm; 12/12: 100%) were better than those using 7ATA (PCO:PO_2_ = 4:3 atm; 9/12: 75%) on preserved rat hearts for 48 h with HPG preservation (Table 2). It is well known that high pressure causes organ damage because decompression injury and excessive CO and O_2_ are highly toxic to preserved organs. Therefore, the use of the appropriate pressure is important for HPG preservation methods.

It was also found that either CO or O_2_ alone could not preserve rat hearts for 48 h under high-pressure CO (PCO = 7 atm), O_2_ (PO_2_ = 7 atm), CO (PCO = 3.5 atm), O_2_ (PO_2_ = 3.5 atm), CO (PCO = 1 atm), and O_2_ (PO_2_ = 1 atm) groups (Table 2). In regard to this combining (partial pressure) of CO and O2, surprisingly, in the present study, CO + O_2_ (PCO:PO_2_ = 1.5:2.0 atm) reduced infarct area after 24 h preservation and improved the survival rate of heart grafts after 48 h preservation compared with CO + O_2_ (PCO:PO_2_ = 2.0:1.5 atm). Furthermore, the mRNA expression of the proinflammatory cytokines TNF-α and IL-1β was also significantly higher in the CO + O_2_ (PCO:PO_2_ = 2.0:1.5 atm) group than in the CO + O_2_ (PCO:PO_2_ = 1.5:2.0 atm) group (Fig. 2). These cytokines could stimulate NF-κB, a key transcription factor in the regulation of IRI, resulting in increased expression of apoptosis-related proteins caspase 3, 8, and 9. The increase of iNOS, a marker of oxidative stress, in the CO + O_2_ (PCO:PO_2_ = 2.0:1.5 atm) group may be also implicated in the pathophysiology of heart IRI. These results indicated that the effects of CO, such as anti-inflammation and anti-apoptosis, depend on the ratio of CO and O_2_. Optimising the ratio of these gases could reduce the side effects of oxidative stress and IRI. Further investigations to identify the molecules associated with CO and O_2_ will help elucidate the optimal conditions for HPG preservation.

We confirmed differences in partial pressures of CO and O_2_ using the Langendorff system as an *ex vivo* evaluation (Fig. 3) and found that the results were similar to those using heterotopic cervical heart transplantation (Table 2). Moreover, the CO3.5-mosit (PCO:PO_2_ = 1.5:2.0 atm) and CO7-moist (PCO:PO_2_ = 4:3 atm) groups showed the highest survival rates and also showed similar trends in the analysis of the heterotopic cervical heart transplantation (Table 2) and Langendorff perfusion system (Supplementary Fig. S1). Further studies using orthotopic transplantation in large animals are required to better understand the detailed function of preserved hearts.

In conclusion, exposure of rat hearts to hyperbaric CO and O_2_ without immersion in CS solution is effective to obtain the necessary cell-protective effects of gas for preservation. To date, many studies have reported the superiority of CS compared with machine perfusion; however, both methods were developed to expand the donor source, and these are not conflicting concepts. A protocol combining the advantages of CS and machine perfusion as well as other methods is required, and HPG using cell-protective gases may allow further expansion for organ preservation.

## Methods

### Animals

An inbred line of LEW/SsN Slc rats (male, 10 weeks old; average weight, 230 g; range, 220–245 g; intact, n = 4; donors, n = 165; recipients, n = 165) was purchased from the Shizuoka Laboratory Animal Center (Shizuoka, Japan). Handling and care of the rats conformed to the National Institutes of Health guidelines for animal research, and all experimental protocols involving animals were approved by the Committee for Animal Care at Aichi Medical University (Permit No. 2016-11, 2017-46, 2018-70). All experiments involving animals were performed in accordance with the relevant guidelines and experimental protocols. Every effort was made to minimise animal suffering.

### HPG preservation method

**Figure 4:**
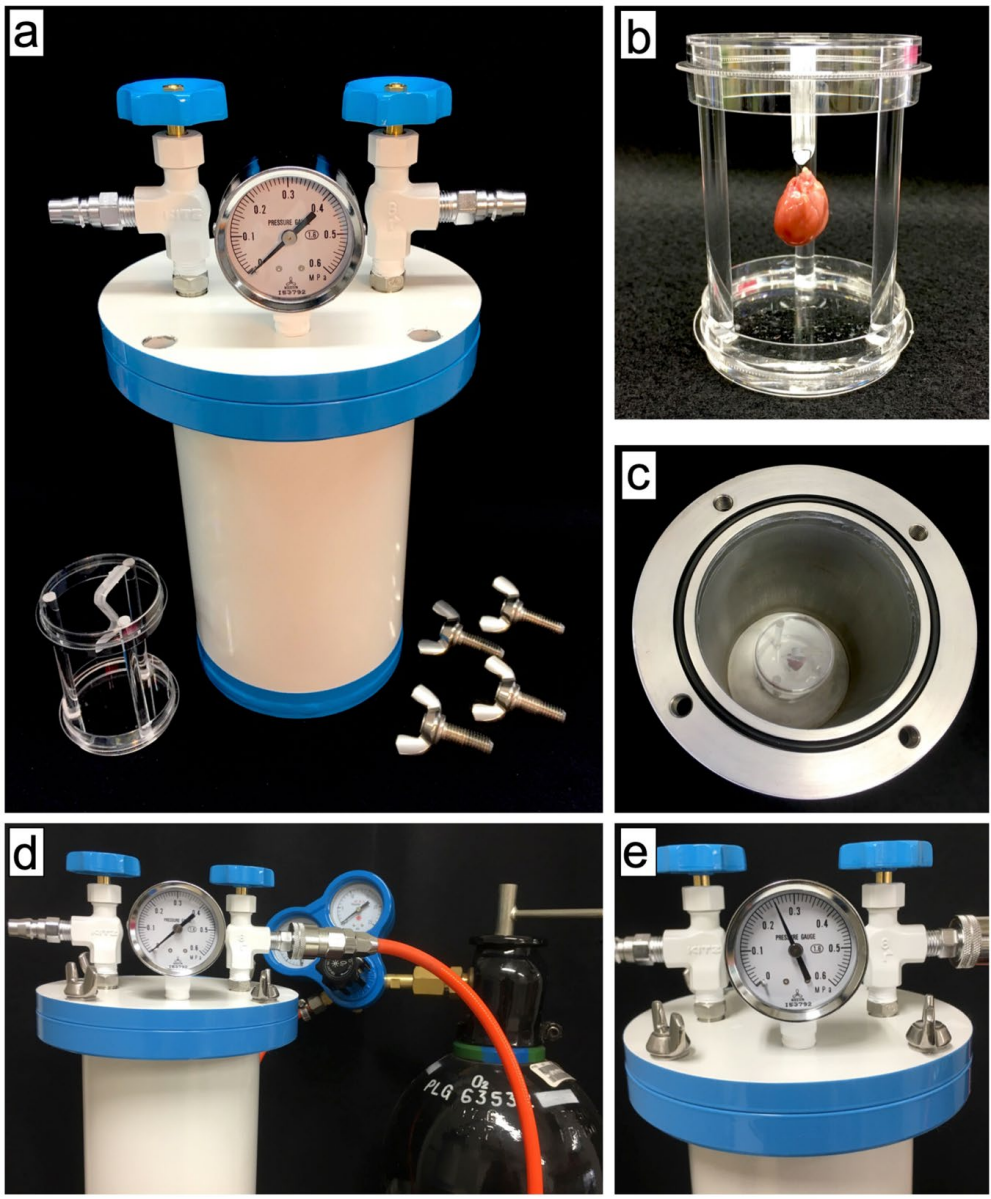
Photos of the HPG preservation method. (**a**) A 7 atm pressure-resistant chamber. (**b**) Extracted rat heart suspended in a plastic cylinder with water to maintain humidity. (**c**) Lumen of a chamber, into which a rat heart was placed. (**d**) Before filiation with CO and O_2_. (**e**) After filiation with CO and O_2_.

For the HPG preservation method, we developed a chamber that could withstand high pressure (Nakamura Iron Works Co., Ltd. Tokyo, Japan) (Fig. 4). To prepare for organ preservation, the inner chamber was placed in distilled water to maintain humidity and then cooled to 4 °C. Extracted rat heart suspended in a plastic cylinder was placed in the chamber, and the lid was closed using four bolts. The chamber was filled with preservation gases. The inner chamber gas temperature was initially increased temporarily (peak 16.6 °C ± 0.8 °C) and the temperature was then decreased over approximately 80 s by placing the chamber in a fridge at 4 °C. Using this temperature alteration, the inner cardiac cavity temperature was increased by approximately 1.98 °C ± 0.53 °C (Supplementary Fig. S2). After preservation, the preserved heart was removed, and the gases were released from the chamber.

### Evaluation of preserved rat hearts by heterotopic cervical heart transplantation

Heterotopic cervical heart transplantation was performed as described previously by Heron *et al*.^19^. Briefly, hearts were extracted from male, 10-week-old donor rats (n = 165) under deep anaesthesia using pentobarbital (50 mg/kg; Kyoritsu Seiyaku Corporation, Tokyo, Japan). Blood was removed using Krebs–Henseleit solution following aortic/pulmonary artery incision and further infusion. Male, 10-week-old recipient rats (n = 165) were deeply anaesthetised with pentobarbital (65 mg/kg body weight) and kept under anaesthesia using isoflurane (ISOFLU, Dainippon Sumitomo Pharma Co., Ltd., Osaka, Japan) in the inhalation anaesthesia apparatus (Univentor 400 Anesthesia Unit, Univentor Ltd., Zejtun, Malta). Preserved donor rat hearts were transplanted into the recipient’s cervical region. The aortic arch and pulmonary artery of the donor heart were overlaid at the end of the common carotid artery and external jugular vein with the cuff of the recipients and then fixed to each cuff with a circular silk ligature. The skin was sutured after the heart rate stabilised. The heart rate was observed to be palpating in the neck, and post-transplant hearts were examined by electrocardiogram. Absence of a waveform for 15 min was defined as cardiac arrest.

### Estimation of myocardial infarct size

The size of the myocardial infarction in the three groups [control group, n = 6; CO + O_2_ (PCO:PO_2_ = 2.0:1.5 atm) group, n = 6; and CO + O_2_ (PCO:PO_2_ = 1.5:2.0 atm) group, n = 6] was estimated by TTC staining. Briefly, after reperfusion, hearts were weighed and cut into 2-mm-thick slices vertical to the atrioventricular groove. The slices were stained by incubating in 2% TTC solution in phosphate buffer (0.1 M) at 37 °C for 15 min and then fixed in 4% paraformaldehyde solution. Infarct size (unstained by TTC) was measured by planimetry using Image Pro Plus 5.0 software (Media Cybernetics, Rockville, MD, USA) and was expressed as a percentage of the total heart.

### Light microscopy

Rat hearts (n = 18) from each group were fixed in 10% formalin (3.7% formaldehyde in water) for 3 h. The samples were then washed, dehydrated using an ethanol series, and embedded in paraffin. Serial 6 μm sections were cut using a microtome and evaluated by Masson’s trichrome staining.

### Gene expression analysis

Hearts were obtained from each group at 90 min after transplantation [control group, n = 6; CO + O_2_ (PCO:PO_2_ = 2.0:1.5 atm) group, n = 6; and CO + O_2_ (PCO:PO_2_ = 1.5:2.0 atm) group, n = 6]. Total RNA was isolated from a sample of heart using a TRIzol RNA extraction kit (Invitrogen, CA, USA) according to the manufacturer’s instructions. cDNA was prepared according to a standard protocol (high-capacity cDNA archive kit; PE Applied Biosystems, Foster City, CA, USA) and stored at 80 °C until analysed. Quantitative RT-PCR of the cDNA was performed using a validated SYBR Green gene expression assay along with the SYBR Premix Ex Taq II (TaKaRa Bio Inc., Ohtsu, Japan) to measure rat HO-1; caspases 3, 8, and 9; iNOS; NF-kB, TNF-α, IL-1β, and glyceraldehyde-3-phosphate dehydrogenase (GAPDH). All the primers used are listed in Table 3. Quantitative RT-PCR was performed in duplicate using a thermal cycler dice real-time system TP800 (TaKaRa), and data were analysed using the same system. The comparative Ct method (2^−ΔΔCt^) was used to quantify gene expression levels. Data for quantitative RT-PCR products were standardised to GAPDH, which was used as intact hearts. To confirm the specific amplification of the target genes, each gene product was further separated on a 1.5% agarose gel to detect single bands at the theoretical product sizes, and dissociation curves were analysed to detect single peaks.

**Table 3:** Target gene names, accession numbers, and specific primer pair sequences.

Name	Accession Number	Direction	Sequence 5′ to 3′
IL-1β	NM_031512.2	forward	tgtgatgaaagacggcacac
		reverse	cttcttctttgggtattgtttgg
TNF-α	NM_012675.3	forward	tgtgcctcagcctcttctc
		reverse	gagcccatttgggaacttct
NF-kB	NM_145788.2	forward	ttccccaggaaaggctgt
		reverse	tggttaggaaaaggcttcca
Caspase 3	NM_012922.2	forward	ccgacttcctgtatgcttactcta
		reverse	catgacccgtcccttgaa
Caspase 8	NM_022277.1	forward	agagcctgagggaaagatgtc
		reverse	tcacatcatagttcacgccagt
Caspase 9	NM_031632.1	forward	cgtggtggtcatcctctctc
		reverse	gagcatccatctgtgccata
HO-1	NM_012580	forward	gtcaagcacagggtgacaga
		reverse	ctgcagctcctcaaacagc
iNOS	NM_012611	forward	cccagagtctctagacctcaaca
		reverse	catggtgaacacgttcttgg
GAPDH	M17701.1	forward	agctggtcatcaatgggaaa
		reverse	atttgatgttagcgggatcg

### Langendorff system

Aortas were cannulated and extracted hearts (n = 66) were perfused by retrograde aortic perfusion with Krebs–Henseleit solution (118 mM NaCl, 4.7 mM KCl, 1.8 mM CaCl_2_•2H_2_O, 1.2 mM MgSO4•7H_2_O, 1.2 mM NaH_2_PO_4_•2H_2_O, 25.0 mM NaHCO_3_, and 11.1 mM glucose) was maintained at pH 7.4 by continuously bubbling with 95%:O_2_/5%:CO_2_, and the temperature was maintained at 37 °C. The flow was adjusted once the hearts were mounted on the Langendorff system within the first minute, and flow was maintained between 10 and 15 mL/g wet weight/min using a peristaltic pump (Radnoti LLC., CA, USA). Adequate perfusion was established in this constant flow system as indicated from the aortic perfusion pressure recording. Contractile parameters were measured by insertion of a fluid-filled latex balloon through the left atrium into the left ventricle, connected to a pressure transducer (AD Instruments, UK), and the balloon volume was adjusted to give an end-diastolic pressure of <10 mmHg. Data were continuously recorded using a Power Lab 8 preamplifier/digitiser (AD Instruments, UK).

### Statistical analysis

Statistical analyses were performed using GraphPad Prism 6.0 software (GraphPad Software, CA, USA), and data are presented as the mean ± standard error of the mean. The one-way ANOVA and the two-way ANOVA with post hoc Tukey’s multiple comparison analysis was performed to compare experimental groups. Statistical significance was set at *P* < 0.05.

## Supplementary information

Dataset 1

## Data Availability

All data generated during this study are included in this published article.
