# Supplementary material for: Different effects of partial pressure in a high-pressure gaseous mixture of carbon monoxide and oxygen for rat heart preservation
Source: Sci Rep. 2019 May 16;9:7480. doi: 10.1038/s41598-019-43905-0 (PMC6522590; doi:10.1038/s41598-019-43905-0)
Supplement: Supplementary file 1 — Dataset 1 [file 41598_2019_43905_MOESM1_ESM.pdf]

SUPPLEMENTAL MATERIAL

Supplementary Figure S1
and
Supplementary Figure S2

Different effects of partial pressure in a high-pressure gaseous mixture of carbon monoxide and oxygen for rat heart preservation

Naoyuki Hatayama[1, 2], *Shuichi Hirai[1], Kaori Fukushige[1], Hiroki Yokota[1],
Masahiro Itoh[2], and Munekazu Naito[1]

[1] Department of Anatomy, Aichi Medical University, Aichi, Japan
[2] Department of Anatomy, Tokyo Medical University, Tokyo, Japan
**Corresponding author:* Shuichi Hirai, e-mail: shinamon611@gmail.com

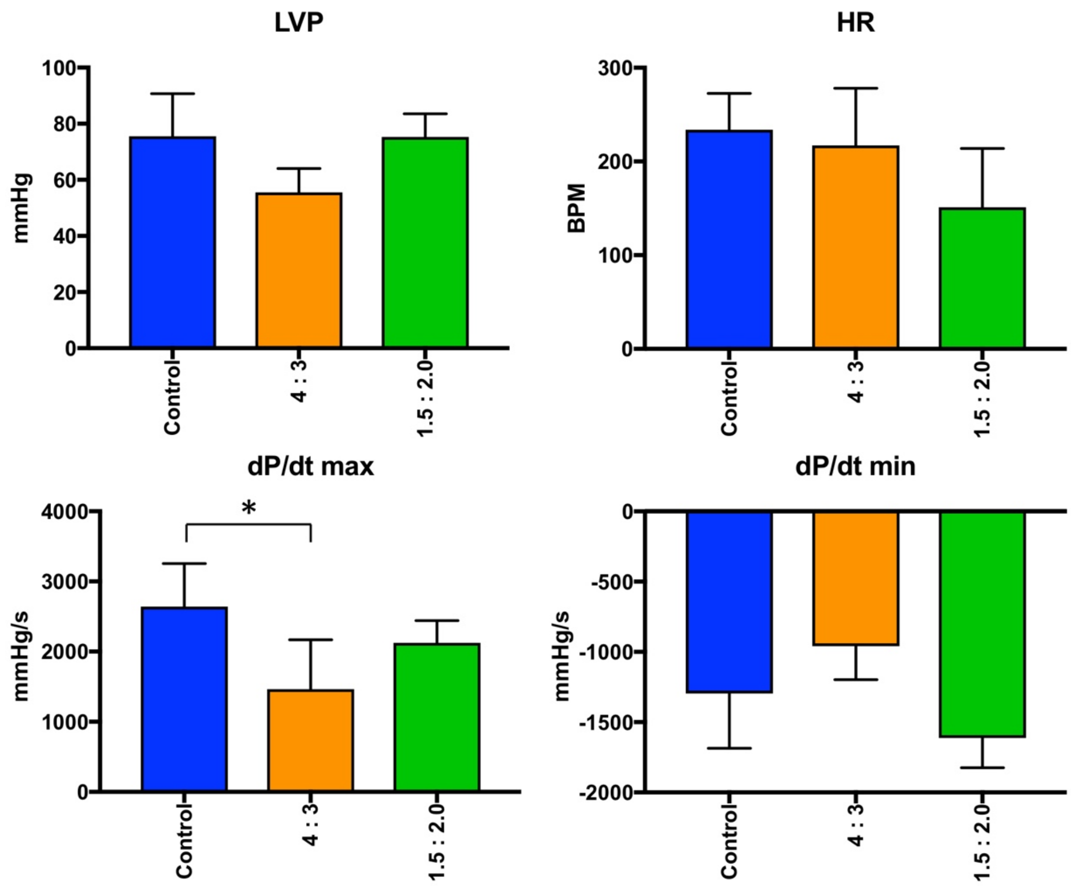

Supplementary Figure S1. Cardiac function after 24-h preservation under conditions of CO + O_2 (PCO:PO_2 = 4:3 atm) and CO + O_2 (PCO:PO_2 = 1.5:2.0 atm) using the Langendorff system.

Hearts were harvested from the control group was rats and immediately perfuse using the Langendorff system (n = 6). LVP, left ventricular pressure; HR, heart rate; dP/dt max and min, peak pressure increase and decrease; N.S., not significant. *$P < 0.05$. *P*-values were calculated by one-way ANOVA with post hoc Tukey's multiple comparison analysis.

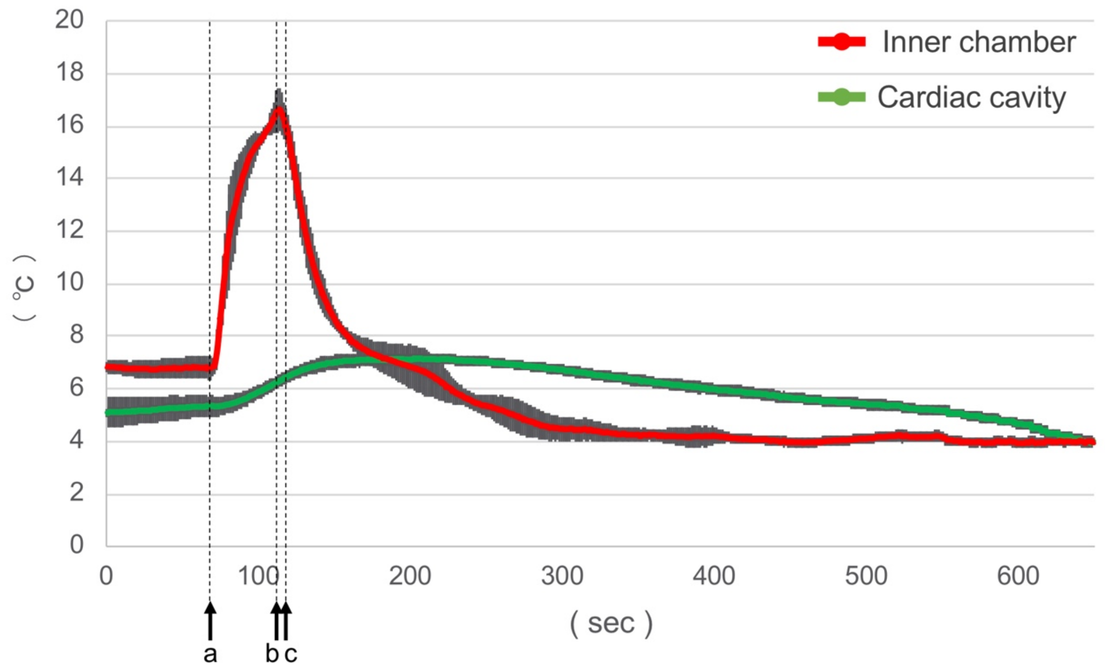

Supplementary Figure S2. Temperature alteration of the inner chamber and cardiac cavity during filling the chamber with gases.

(a) Start of gas filling. (b) End of gas filling. (c) Point at which the chamber was refrigerated.
